# Transcriptomic Analysis of High-Intensity Interval Training in High-Fat-Diet-Induced Spontaneous Hypertensive Rats’ Brains

**DOI:** 10.3390/ijms27010304

**Published:** 2025-12-27

**Authors:** Arslan Sadiq, Iqbal Ali Shah, Bor-Tsang Wu, Yi-Yuan Lin, Yi-An Su, Ai-Lun Yang, Shin-Da Lee

**Affiliations:** 1 Graduate Institute of Acupuncture Sciences, China Medical University, Taichung 404328, Taiwan; 2 PhD Program in Healthcare Science, China Medical University, Taichung 406040, Taiwan; 3 Department of Senior Citizen Service Management, National Taichung University of Science and Technology, Taichung 404336, Taiwan; 4 Department of Exercise and Health Science, National Taipei University of Nursing and Health Sciences, Taipei 11219, Taiwan; 5 Institute of Sports Sciences, University of Taipei, Taipei 11153, Taiwan; annayiansu@gmail.com; 6 Department of Physical Therapy, China Medical University, Taichung 406040, Taiwan

**Keywords:** apoptosis, brain cortex, exercise training, gene analysis, hypertension

## Abstract

Hypertension contributes to brain dysfunction through apoptosis, oxidative stress, reduced neuronal connectivity, and neurotransmitter imbalance. Exercise training is a non-pharmacological strategy known to modulate these molecular alterations. This study investigated the effects of high-intensity interval training (HIIT) on transcriptomic changes in the cerebral cortex of spontaneously hypertensive rats (SHR) fed a high-fat diet (HFD). Rats were assigned to either a HIIT intervention group (HIIT-HFD-SHR) or a sedentary control group (HFD-SHR). Cortical RNA was extracted, sequenced using the Illumina NovaSeq 6000 platform, and analyzed with DESeq2. Functional enrichment was conducted using Metascape. RNA-seq identified 1223 differentially expressed genes (DEGs) (adjusted *p* < 0.05), with 51 remaining significant under stringent criteria (adjusted *p* < 0.001, |log_2_FC| > 0.5). Among these, eight key genes were closely associated with the regulation of apoptosis and autophagy, including seven downregulated (*Egr1*, *Atf3*, *Tgm2*, *Lgals1*, *Nr4a1*, *Plekhf1*, *Nupr1*) and one upregulated (*Trim39*). This transcriptomic analysis following HIIT also modulated circadian rhythm, long-term memory processes, and hypoxia response in the hypertensive brain. These findings indicate that HIIT decreases apoptosis and autophagy and improves circadian rhythm, long-term memory, and hypoxia in hypertensive rats’ brains.

## 1. Introduction

Hypertension is one of the most serious medical conditions that badly affects all vital organs, including the brain cortex [1,2]. Disruptions in blood pressure affect cognitive impairment in the patient’s brain [3]. In particular, a recent study found that systolic blood pressure had a high correlation with abnormal changes in the hypertensive brain’s physiology [4]. The brain’s physiology changes drastically during hypertension, such as a decrease in cell number, cortical volume, and neuronal connections [5]. Several experimental models of hypertension have been created to replicate human hypertension, with the spontaneously hypertensive rat (SHR) being a widely used model [6].

High-fat diet (HFD) reportedly has a high correlation with dementia development due to insulin disruption [7]. HFD affected cortex samples by increasing oxidative stress, which potentially affected neuronal health and function [8]. In addition, HFD had significant association with neurotransmitter alteration in the prefrontal cortex [9]. As a consequence, HFD is involved in hypertension and contributes to brain damage through neuron component imbalance [10]. HFD rat models can be achieved either through genetic or diet manipulation. C57BL/6J mice, Sprague-Dawley, and Wistar rats are frequently used for studies involving HFD-induced obesity combined with hypertension [6].

Transcriptomic analysis provides key insights into differentially expressed genes and the pathways they activate or suppress, helping to elucidate molecular mechanisms at the transcriptome level. Cell death in the brain cortex could be caused by hypertension and high-fat diet consumption [11,12]. Hypertension caused cell death in the brain through oxidative stress and other pathways [13]. Unfortunately, the detailed neural degeneration mechanism during hypertension is still under investigation [14]. Meanwhile, the HFD effect on cell death in the brain occurs because of autophagy disruption [15].

Exercise can reportedly stimulate neuronal cells and increase cognitive ability [16]. Furthermore, exercise decreased hypertension symptoms and reduced HFD effects [17,18]. Exercise decreased fat composition and lipid metabolism in HFD-consuming mice [19]. On the other hand, exercise contributes to alleviating vasoconstriction, which usually happens in hypertension [20]. In the end, exercise has the potential to be a treatment for neuronal diseases caused by hypertension and HFD. High-intensity interval training (HIIT) has become a well-established approach to increasing functional capacity in patients with hypertension [21]. HIIT combines brief, high-intensity exercise bouts (85–95% of VO_2_max) with lower-intensity recovery periods (50–60% of VO_2_max) [22].

Although the beneficial effects of exercise on cardiovascular and metabolic health are well-recognized [23], the molecular mechanisms underlying its impact on the brain remain unclear. In particular, how exercise regulates whole-genome expression in the cortex under conditions of hypertension and high-fat diet (HFD) is poorly understood. Previous studies have largely focused on peripheral tissues, such as skeletal muscle and the heart [24], leaving cortical adaptations largely unexplored. Even where brain tissue has been examined, most work has targeted the hippocampus or prefrontal cortex in diet-induced obesity models without considering hypertension or high-intensity interval training [25]. The specific signaling pathways and gene networks modulated in this disease context have not been systematically characterized. This gap underscores the need to investigate cortical transcriptomic regulation to clarify how high-intensity interval training confers neuroprotection in hypertension with HFD.

## 2. Results

The high-intensity interval training high-fat diet (HIIT-HFD) spontaneously hypertensive rat (SHR) group (*n* = 4) exhibited a mean body weight of 341.8 ± 15.3 g and a mean blood pressure of 140.3 ± 17.7 mmHg, with a mean heart rate of 371.3 ± 17.3. In contrast, the sedentary high-fat diet (HFD) SHR group (*n* = 4) had a slightly higher mean body weight of 361.7 ± 26.1 g, *p* ≤ 0.11383, and a markedly elevated mean blood pressure of 155 ± 6.6 mmHg, with a mean heart rate of 415.8 ± 9.2. This randomized control trial was conducted to explore the effect of high-intensity interval training (HIIT) on hypertensive rats. Transcriptomic analysis revealed that 1223 significant genes were differentially expressed in the HIIT-HFD rats compared to sedentary rats. The significant value was *p* ≤ 0.05.

**Figure 1:**
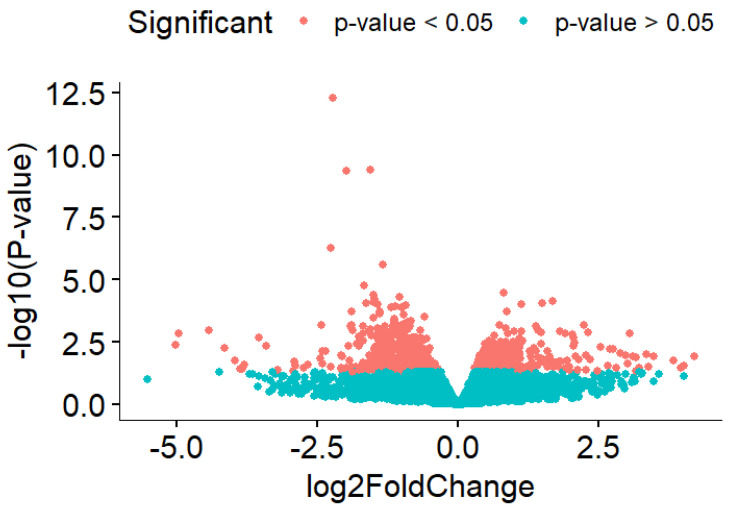
Volcano plot that shows significantly differentially expressed genes between the HIIT HFD-SHR rat and the sedentary HFD-SHR rat.

Due to massive amounts of genes being significant in HIIT activity compared to the sedentary group, a stricter filter was applied by increasing the *p*-value filter from 0.05 to 0.001 and absolute logFC to more than 0.5. The strict filter (*p*-value < 0.001 and absolute logFC > 0.5) resulted in 51 significant genes, with 9 genes being upregulated and 42 genes being downregulated in HIIT-HFD-SHR samples compared to the sedentary HFD-SHR samples (Figure 1).

**Figure 2:**
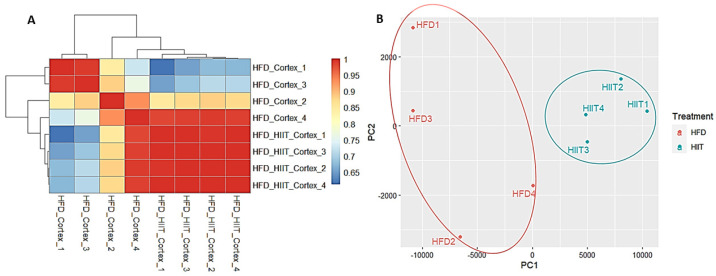
Sample quality between HFD-SHR and HIIT-HFD-SHR samples. (**A**) Heatmap. (**B**) PCA analysis.

The sample quality visualization with a heatmap and PCA showed that HIIT-HFD-SHR samples had different characteristics compared to the sedentary HFD-SHR samples. Hierarchical clustering and PCA of the RNA-seq data were conducted to evaluate potential technical variability and batch effects. Both analyses demonstrated clear separation of HFD and HIIT samples, with biological replicates clustering tightly within groups, confirming that batch effects were negligible, as shown in Figure 2. Sample quality was based on *p*-value < 0.001 and absolute logFC > 0.5, which resulted in 51 significantly differentially expressed genes. Based on differentially expressed genes and sample quality analysis, these 51 significant genes were analyzed further by performing functional enrichment analysis.

### Differentially Expressed Gene Pathway via Functional Enrichment Analysis

**Figure 3:**
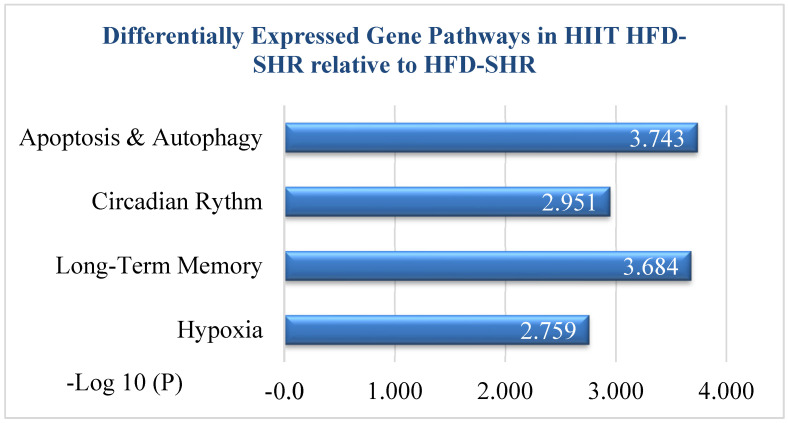
Functional enrichment analysis based on Metascape showed that positive regulation of the apoptotic process reached the top 4 most significant functional enrichments in HIIT-HFD-SHR samples compared to HFD-SHR samples. The *x*-axis indicates enrichment significance, expressed as −log10 (*p*-value). The *y*-axis indicates enriched pathways.

Functional enrichment analysis, which used Metascape software, V.3.5 showed that various pathways and biological processes were significant (Figure 3). The cell-death-related biological process was significant based on the GOBP database. “Positive regulation of apoptotic process” enrichment was significant and reached the top three most significant pathways in HIIT-HFD-SHR samples compared to sedentary HFD-SHR samples. As mentioned in Table 1, given below, there were eight most significantly differentially expressed genes in the apoptotic pathway; among them, seven were downregulated and one was upregulated (*Trim39*). Atf3 was most significant among the seven downregulated genes.

Secondly, functional enrichment analysis based on Metascape shows circadian rhythm in HIIT-HFD SHR samples compared to HFD-SHR samples, and there were four significant genes that were expressed, given as *EGR1*, *Per2*, *Per3*, and *Adcy1*. Thirdly, the functional enrichment analysis based on Metascape shows long-term memory, and it shows the three most significantly expressed genes as *Egr1*, *Arc*, and *Adcy1*. Lastly, hypoxia is shown through functional enrichment analysis based on Metascape, and there were four most expressed genes, given as *Egr1*, *Junb*, *Cox4i2*, and *Fos*.

## 3. Discussion

To our knowledge, this is the first study to demonstrate that high-intensity interval training (HIIT) exerts beneficial effects by reducing apoptosis and autophagy in the brain of high-fat-diet-induced hypertensive rats, and no previous study has combined high-intensity interval training with a high-fat diet in spontaneously hypertensive rats. Therefore, our HIIT–HFD–SHR model provides a novel framework for examining how exercise modifies transcriptomic responses under the dual influence of genetic hypertension and diet-induced metabolic stress.

Recent studies indicate that high-intensity interval training (HIIT) induces brain molecular adaptations, although transcriptomic effects appear more selective than those observed with moderate-intensity continuous training (MICT). Marcourt et al. (2025) reported broader hippocampal and cortical transcriptomic remodeling with MICT in aged Wistar rats, whereas HIIT elicited targeted gene expression changes with enhanced mitochondrial and cardiovascular function [26]. Similarly, Khoramipour et al. (2023) showed that HIIT attenuated hippocampal molecular dysfunction in high-fat-diet diabetic rats, despite not employing transcriptome-wide analysis. Importantly, transcriptomic evidence for HIIT in hypertensive models remains lacking [27]. In this context, our study is the first to systematically characterize HIIT-induced transcriptomic remodeling in the cerebral cortex of spontaneously hypertensive rats under high-fat-diet conditions, providing novel insight into cortex-specific gene networks associated with metabolic regulation and neuroplasticity.

**Figure 4:**
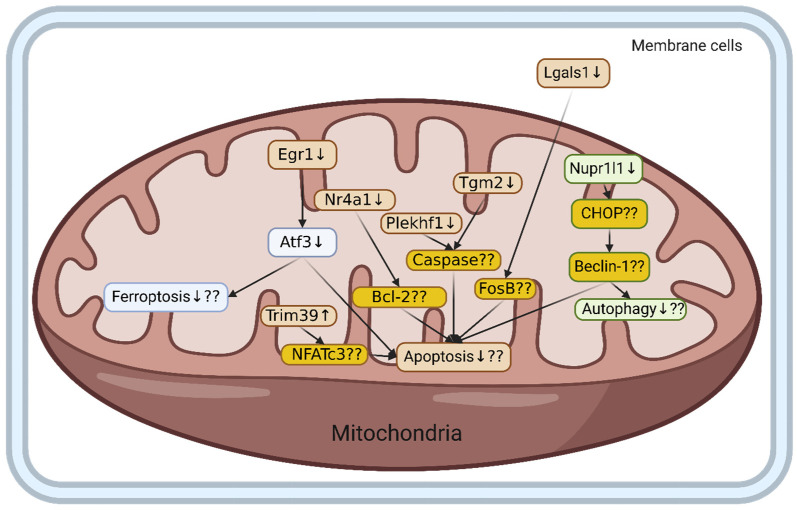
The potential effects of HIIT exercise on ell death in hypertensive cortex condition with high fat diet. Hypothetical pathway underlying HIIT-mediated modulation of cortical pathology in HFD hypertensive rats. Genes upregulated ↑, genes downregulated ↓, genes’ regulation not confirmed ??, dark yellow = cell-death-related genes, ?? = requires further investigation.

Our transcriptomic analysis identified 1223 significantly differentially expressed genes (DEGs) following HIIT intervention. Functional enrichment analysis revealed 51 highly significant DEGs, including 9 upregulated and 42 downregulated genes. Among these eight key genes, seven of these genes, *Egr1*, *Atf3*, *Tgm2*, *Lgals1*, *Nr4a1*, *Plekhf1*, and *Nupr1*, were downregulated, while Trim39 was upregulated following HIIT. KEGG pathway analysis indicated that genes involved in the positive regulation of cell death were predominantly downregulated, suggesting that HIIT may attenuate apoptosis and promote neuroprotection under conditions of high-fat-diet-induced hypertension (Figure 4).

The experimental study showed that high-intensity interval training in hypertension fed with a high-fat diet could reduce cell-death-related genes in animals [28]. This study found that high-intensity interval training had potential in reducing cell death in hypertensive rats fed a high-fat diet because any genes related to positive cell death regulation were downregulated. Interestingly, most of the genes after high-intensity interval training in hypertensive rats fed a high-fat diet were involved in mitochondria. Previous studies have also shown that high-intensity interval training can prevent mitochondrial dysfunction induced by a high-fat diet [29], implying that this study had similar results to previous research.

High-intensity interval training in hypertensive rats fed a high-fat diet had the potential to affect three cell death mechanisms. First, ferroptosis was affected by high-intensity interval training in hypertensive rats fed a high-fat diet through two different genes, *Egr1* and *Atf3*. Previous studies have revealed that *Egr1* was upregulated during hypertensive conditions [30], and high-intensity interval training in hypertensive rats fed a high-fat diet was downregulated during this study.

*Egr1* is involved in cell death in the brain by regulating *Atf3* and the other genes when the brain is disrupted [31]. *Atf3* was upregulated during brain dysfunction, inducing inflammation [32], and it was downregulated by high-intensity interval training in hypertensive rats fed a high-fat diet in this study, implying that high-intensity interval training had alleviated brain disruption. *Atf3* reportedly contributed to ferroptosis by inducing endoplasmic reticulum stress and mitochondria [33], and *Atf3* was downregulated by high-intensity interval training in hypertensive rats fed a high-fat diet in this study.

Apoptosis was the second cell death mechanism that was affected by high-intensity interval training during the HFD hypertensive cortex condition. There were several genes affected by high-intensity interval training during HFD hypertension, which were *Trim39*, *Nr4a1*, *Plekhf1*, *Tgm2*, *Lgals1*, *Egr1*, and *Atf3*. *Trim39* regulated neuron apoptosis, because when this gene was knocked out, neuronal apoptosis increased through *NfAtc3*, while high-intensity interval training increased *Trim39* expression, indicating that high-intensity interval training potentially reduced apoptosis because *Trim39* was upregulated [34]. *Nr4a1* induced apoptosis by interacting with *Bcl-2* in the mitochondria, which led to neuronal cell death, while high-intensity interval training downregulated *Nr4a1*, which had the potential to reduce the apoptosis rate in HFD hypertensive cortex samples [35]. *Plekhf1* upregulation contributed to the apoptosis process in the mitochondria through the caspase-related process [36], and high-intensity interval training downregulated *Plekhf1* expression. As such, high-intensity interval training might have the potential to reduce the apoptosis process. *Tgm2* upregulation contributed to apoptosis through caspase-related genes, but its contribution to *Plekhf1* is still unknown [37]. However, *Tgm2* was downregulated with high-intensity interval training in the HFD hypertensive cortex, which might lead to a reduced apoptosis rate. *Lgals1*’s high expression, which was in membrane cells, influenced immune response and apoptosis through the Fos gene [38]. *Lgals1* expression was downregulated by high-intensity interval training, which might have the potential to reduce the apoptosis rate during HFD hypertension in the cortex. *Egr1* and *Atf3* reportedly also contribute to apoptosis, which might have dual effects, because those genes also contributed to ferroptosis [31]. *Egr1* and *Atf3* were downregulated by high-intensity interval training in hypertensive rats fed a high-fat diet in this study.

Furthermore, our study showed that high-intensity interval training regulates autophagy through *beclin-1*, and these results are consistent with previous findings in diabetic hearts, where high-intensity interval training regulated *beclin-1* and other autophagy-related genes, further supporting high-intensity interval training as a good modulator of metabolic stress and tissue health and its key role in regulating cellular repair mechanisms [39].

Autophagy was potentially influenced by high-intensity interval training as well during hypertensive cortex conditions. The *Nupr II* gene acts as a transcriptional regulator that controls the expression of CHOP and beclin-1. In abnormal conditions, these genes cause autophagy, which is one of the mechanisms of apoptosis [40]. The present study reports downregulation of the *Nupr II* gene after high-intensity interval training in hypertensive rats fed a high-fat diet, thereby preventing apoptosis.

Our study reveals that high-intensity interval training in hypertensive rats fed a high-fat diet restores cortical circadian rhythmicity by significantly downregulating *Egr1*, *Per2*, *Per3*, and *Adcy1* expression. The observed suppression of *Egr1*, a known transcriptional activator of *Per2* in neuronal tissue via the ERK–Elk1 pathway, likely disrupts a key driver of PRR gene overexpression [41]. As observed by Wolff and Esser, forced exercise acts as a zeitgeber through elevated corticosterone entraining *PER2* oscillations in the cortex and other tissues, a mechanism potentially mirrored in our HIIT study [42]. Additionally, rhythmic corticosterone signaling has been shown to modulate *Per2* expression in the prefrontal cortex, suggesting that HIIT-induced endocrine changes contribute to molecular clock realignment [43]. Finally, the downregulation of *Adcy1*, an enzyme crucial for cAMP-CREB signaling and synaptic plasticity, may reflect a normalization of circadian-linked neuronal signaling networks, as highlighted in stress-adapted models [44]. Overall, our results suggest that high-intensity interval training in hypertensive rats fed a high-fat diet reduces the activity of the *Egr1*–Per pathway and adjusts cAMP signaling, which helps reset clock gene activity in the brain and brings back normal daily rhythms [45,46]. These results indicate that consumption of a HFD with hypertension disrupts the circadian clock in animal models and that high-intensity interval training in hypertensive rats fed a high-fat diet has a beneficial effect to restore it.

Our present study demonstrates that high-intensity interval training in hypertensive rats fed a high-fat diet markedly modifies the expression of key long-term memory-related genes: *Egr1*, *Arc*, and *Adcy1*. HFD alone has been shown to diminish hippocampal Arc levels, impairing memory consolidation via insulin-resistance-mediated PI3K/Akt pathway suppression [47]. Previous studies have reported that HIIT upregulates the immediate early genes Arc and Egr1, which are crucial for synaptic plasticity and long-term potentiation (LTP), suggesting reactivation of hippocampal memory circuits [48]. In contrast, in our study, Arc and Egr1 were both downregulated following HIIT in hypertensive rats fed a high-fat diet, indicating a distinct and context-dependent transcriptional response. Concurrently, the normalization of *Adcy1*, encoding calcium-stimulated adenylyl cyclase-1 and essential for cAMP/CREB-driven synaptic strengthening, aligns with prior evidence that *Adcy1* overexpression enhances CREB phosphorylation, LTP, and memory performance [49]. Thus, high-intensity interval training appears to counteract cognitive decline by reinstating a coordinated gene network with Arc and *Egr1* to initiate synaptic transcriptional programs and *Adcy1* to restore intracellular signaling, culminating in improved memory-related neural plasticity [50]. Thus, high-intensity interval training in hypertensive rats fed a high-fat diet appears to counteract cognitive decline via long-term memory-related genes.

Our study reveals that high-intensity interval training significantly alters the expression of hypoxia-related genes *Egr1*, *Junb*, *Cox4i2*, and *cFos*, indicating that high-intensity interval training triggers molecular responses like mild hypoxic conditions in the brain. The upregulation of *Egr1* and *Fos*, classic immediate early genes, mirrors findings where high-intensity exercise boosts *c-Fos* and *Egr1* expression in hippocampal neurons, reflecting neural activation and plasticity [51]. The Junb/AP-1 transcription complex collaborates with *cFos* under hypoxia and stress conditions to regulate genes involved in cellular adaptation [52]. Notably, *Cox4i2*, encoding a hypoxia-responsive cytochrome c oxidase subunit, is known to increase expression during low-oxygen conditions, including sprint exercise in muscle, suggesting a metabolic shift toward hypoxic adaptation [53]. Collectively, these molecular shifts demonstrate that high-intensity interval training in hypertensive rats fed a high-fat diet initiates a coordinated hypoxia-like transcriptional program, potentially enhancing cerebral oxygen utilization, mitochondrial efficiency, and overall neural resilience, an important mechanism through which high-intensity interval training promotes brain health.

There were several limitations to this study. Only young animals at 24 weeks old were recruited for this study, which may not fully represent age-related, obesity-induced hypertension in humans. Treadmill training can be more stressful for animal models compared to humans, which can further affect the outcomes of the present study. This study could not differentiate whether gene expression changes originated from mitochondrial, cytosolic, or nuclear compartments, as the methodology does not allow for subcellular localization of gene expression. Another limitation is the lack of available literature on the effects of high-intensity interval training in hypertensive rats fed a high-fat diet, making it difficult to compare and support our findings. This study was designed as a transcriptomic exploratory analysis with sample sizes consistent with published RNA-seq studies. Future work with larger animal cohorts will be essential to validate the reproducibility and translational relevance of the identified gene expression changes. The transcriptomic data represent a single endpoint following the HIIT intervention; therefore, the temporal stability of these gene expression changes remains to be determined. Future multi-time-point analyses will be required to assess whether these effects are transient or sustained.

## 4. Materials and Methods

### 4.1. Animals

Eight rats were purchased from Bio LASCO Nangang Dist, Taipei city, Taiwan Co., Ltd., and the present study was approved on 24 February 2023 by the International Animal Care and Use Committee (IACUC), approval number UT 112001, University of Taipei, Taipei, Taiwan. The HFD group was provided with the high-fat diet containing 45% fat for 12 weeks (Rodent diet D12451, Research Diets, Inc., New Brunswick, NJ, USA). Twenty-four-week-old male high-fat diet (HFD) spontaneous hypertensive rats (SHR) (*n* = 8) were randomly divided into two groups: an exercise group and a sedentary group. The exercise group received 12 weeks of HIIT on a motorized treadmill, and the sedentary group received active placebo treatment.

### 4.2. Exercise Procedure

Rats in the HIIT group were trained on a treadmill with high-intensity intervals set at 25 m/min (corresponding to 85–90% VO_2_max) for 4 min, followed by low-intensity intervals at 15 m/min (50–60% VO_2_max) for 3 min. This cycle was repeated seven times, resulting in a total session duration of 49 min. Training was conducted 5 days per week for 12 weeks, consistent with previously validated protocols in rodent models [54,55]. Treadmill inclination was increased by 5° after 4 weeks to progressively adjust exercise intensity, in accordance with methods used to maintain relative workload during long-term HIIT in rats [56]. Sedentary control animals were placed on the treadmill without running to ensure equivalent environmental exposure.

To estimate VO_2_max, treadmill speeds were selected based on prior studies that measured oxygen consumption in rats, enabling the application of %VO_2_max for both high- and low-intensity intervals [54,55]. This approach ensures that exercise intensity is standardized and comparable across subjects while maintaining the relative workload throughout the training period.

### 4.3. RNA Isolation

The brain cortex region from controlled and treated rats was surgically isolated under sterile conditions. Tissue samples were immediately processed in the laboratory for RNA extraction. Total RNA was extracted from 50 mg of tissue and homogenized in 500 μL of TRIzol (Thermo Fisher Scientific, Waltham, MA, USA) with steel beads using a TissueLyser II (Qiagen, Hilden, Germany) [57]. Proteinase K (Worthington Biochemical Corporation, Lakewood, NJ, USA) was added to the sample to degrade proteins. Following phase separation with BCP, RNA was precipitated with ethanol and purified using the miRNeasy Mini Kit (Qiagen) per the manufacturer’s instructions. RNA quality and quantity were assessed with a Fragment Analyzer 5200 (Agilent Technologies, Santa Clara, CA, USA) and a Nanodrop (Thermo Scientific, Waltham, MA, USA).

RNA quality and quantity were assessed using a Fragment Analyzer 5200 (Agilent) with the DNF-471 RNA Kit. All samples showed total RNA yields > 2 µg per sample and RNA fragment size distributions within the acceptable analytical range of the kit (5–500 nt). Based on these criteria, all RNA samples met the manufacturer’s quality requirements and were classified as “Pass.” RNA concentration was additionally confirmed using a NanoDrop spectrophotometer (Thermo Scientific, Waltham, MA, USA).

### 4.4. Transcriptomic Analysis

Transcriptomic analysis refers to the comprehensive examination of the transcriptome. The complete set of RNA transcripts expressed in a cell or tissue at a given time is analyzed using high-throughput sequencing technologies, such as RNA-seq. It provides both qualitative and quantitative insights into gene expression dynamics, enabling the identification of differentially expressed genes, alternative splicing events, and functional pathways under specific physiological or experimental conditions [58].

### 4.5. RNA-Sequencing and Data Analysis

RNA libraries were prepared using the TruSeq Stranded mRNA Library Prep Kit (Illumina, San Diego, CA, USA) according to the manufacturer’s instructions [59]. mRNA was isolated from total RNA (1 µg) using oligo(dT)-coupled magnetic beads and fragmented at high temperature. First-strand cDNA was synthesized using reverse transcriptase and random primers, followed by second-strand cDNA synthesis. Adaptors were ligated to the double-stranded cDNA, and adenylation was performed at the 3′ ends of DNA fragments. Libraries were amplified via PCR and purified using the AMPure XP system (Beckman Coulter, Brea, CA, USA).

Library size distribution was evaluated using the Qsep400 System (Bioptic Inc., New Taipei City, Taiwan), and library concentration was quantified using a Qubit 2.0 Fluorometer (Thermo Scientific, Waltham, MA, USA). Library quality control followed a standardized and documented sequencing workflow (Library QC, Version 2023_V1, June 2023). Only libraries meeting quality control criteria were sequenced. High-quality libraries were sequenced on an Illumina NovaSeq platform using 150 bp paired-end reads, achieving adequate sequencing depth and read quality for transcriptomic analysis.

Library quality was assessed using the Qsep400 System (Bioptic Inc., Taiwan) and quantified with a Qubit 2.0 Fluorometer (Thermo Scientific, USA). Qualified libraries were sequenced on an Illumina NovaSeq platform using 150-bp paired-end reads (Genomics, BioSci & Tech Co., New Taipei City, Taiwan).

Base call (BCL) files were converted to FASTQ format using bcl2fastq (v2.20). Adapters and low-quality reads (reads below Q30) were trimmed using fastp (v0.23.2). Clean reads were aligned to the mouse reference genome (GRCm38/mm10) using STAR (v2.7.9a) with default parameters. Gene-level counts were obtained using feature counts (v2.0.1). Differential expression analysis was conducted using DESeq2 (v1.34.0), with genes showing an adjusted *p*-value < 0.001 (Benjamini–Hochberg correction) and |log_2_ fold change| > 0.5 considered significant [60]. Functional enrichment analysis of differentially expressed genes was performed using cluster Profiler (v4.0.5) against the KEGG and Gene Ontology databases, with *p*-value < 0.05 used as the significance threshold. R language was applied in differential gene expression analysis. Metascape was applied for functional enrichment analysis [61].

## 5. Conclusions

This transcriptomic analysis identified 1223 differentially expressed genes in the cerebral cortex of high-fat-diet-induced spontaneously hypertensive rats following high-intensity interval training (HIIT). Among these, eight key genes, *Egr1*, *Atf3*, *Tgm2*, *Lgals1*, *Nr4a1*, *Plekhf1*, *Trim39*, and *Nupr1*, were significantly associated with the regulation of apoptosis and autophagy. HIIT effectively modulated pathways related to circadian rhythm, hypoxia-induced neural activation, and long-term memory, suggesting its potential therapeutic role in promoting neuroprotection in hypertensive brains.

## Figures and Tables

**Table 1:** Positive regulation of apoptotic process; those genes were mostly downregulated. Fold change (FC), significant value (*p* ≤ 0.05).

#	Symbol	log_2_FC	*p* Value
1	Egr1	−0.845	<0.001
2	Atf3	−1.683	0.001
3	Tgm2	−0.965	0.001
4	Lgals1	−1.085	0.001
5	Nr4a1	−1.423	<0.001
6	Plekhf1	−0.891	0.001
7	Trim39	1.496	<0.001
8	Nupr1l1	−1.504	<0.001

Positive regulation of apoptotic process corresponding genes. (1) Early growth response 1, (2) Activating transcription factor 3, (3) Transglutaminase 2, (4) Galectin-1, (5) Nuclear receptor subfamily 4 group A member 1, (6) Pleckstrin homology domain containing family F member 1, (7) Tripartite motif containing 39, and (8) Nuclear Protein1, as shown in Table 1.

## Data Availability

Data are presented in the manuscript. Some data will be provided upon special request to the corresponding authors.
